# Evaluation of Biocompatible and Biodegradable PES/PCL Membranes for Potential Use in Biomedical Devices: From Fouling Resistance to Environmental Safety

**DOI:** 10.3390/molecules30193887

**Published:** 2025-09-25

**Authors:** Cezary Wojciechowski, Monika Wasyłeczko, Dorota Lewińska, Andrzej Chwojnowski

**Affiliations:** Nałęcz Institute of Biocybernetics and Biomedical Engineering, Polish Academy of Science, 02-109 Warsaw, Poland; dlewinska@ibib.waw.pl (D.L.); achwojnowski@ibib.waw.pl (A.C.)

**Keywords:** capillary membranes, biomaterials, biocompatibility, synthetic polymers, polymer-based materials, medical devices, retention, degradation, hydrolysis, fouling

## Abstract

The paper presents a method for obtaining partially degradable capillary membranes from a polyethersulfone/polycaprolactone (PES/PCL) mixture. PES/PCL membranes were obtained by the phase inversion technique with dry/wet spinning and then subjected to controlled degradation in an alkaline environment (1 M NaOH) and simulated body fluid (SBF with pH 7.4) using the flow method. The aim of the work was to select and apply a degradable, non-toxic, simple polymer as a removable component of the membrane structure. The degradable component of the membranes was PCL, the gradual hydrolysis of which was aimed at increasing the porosity and improving the transport properties of the membranes during operation. The membrane properties, such as hydraulic permeability coefficient (UFC), retention coefficient, and structural morphology, were assessed using scanning electron microscopy (SEM) before and after degradation. Analysis of SEM images performed with MeMoExplorer^TM^ software showed an increase in the proportion of large pores (above 300 µm^2^) and total porosity of the membranes after degradation in NaOH and SBF. Low instability factor (<0.25) for all samples, both before and after degradation, confirms the good repeatability of the membrane structure. An increase in the UFC was observed, while the retention coefficients did not change significantly in the case of membranes after the etching process. The degradation of the PCL component in the membrane was assessed using the weight method. Measurements of the membrane mass loss before and after degradation confirmed the removal of over 50 wt.% of the PCL component in SBF and 70 wt.% in NaOH from the tested membranes, which resulted in an increase in permeability due to increased membrane porosity. The results indicate the possibility of using such structures as functional, partially self-regulating membranes, potentially useful in biomedical and environmental applications.

## 1. Introduction

During membrane filtration, there is a decrease in the performance and lifetime of the membrane during its operation, which is a consequence of the accumulation of suspended or dissolved solid particles present in the filtered solution on the surface and/or in the pores of the membrane. This process is called membrane fouling. It reduces the filtration time, which depends on the type of medium used and the characteristics of the membrane itself [1,2].

Currently, there are many techniques for controlling and limiting fouling that extend the useful life of membranes and reduce operating costs. These include all physical treatments, such as periodic backwashing of membranes, optimization of membrane filtration process parameters, as well as chemical treatments, including the use of cleaning agents (NaOCl, NaOH, HCl, HNO_3_). The effectiveness of backwashing depends on the nature of the fouling mechanism, but in the case of clogging and adsorption inside the pores, this method is ineffective, and the use of chemicals is required. The selection of membranes with a lower tendency to foul is also a strategy for controlling this process. Both physical and chemical methods of fouling removal cause interruptions in membrane processes, increase their duration, and raise their costs [3,4,5,6].

Our proposed solution to mitigate the effects of fouling is to use partially biodegradable membranes. Such membranes will undergo slow degradation (hydrolysis) during operation in an aqua environment, which will result in an increase in their porosity and pore size as a result of part of their material being washed out of the membrane structure [7]. This process will happen more in an alkaline or acidic environment, which helps with hydrolysis, and also in the body fluids of living organisms, which have active enzymes that can also affect the structure of these membranes. When fouling occurs during membrane operation, causing pore blockage, the membrane will simultaneously degrade, becoming looser in structure as a result of some of its material being washed out from inside. In this way, the simultaneous processes of fouling and membrane degradation will balance each other out. As a result, the membrane will be able to perform its function for a much longer period. The progressive decline in membrane performance will be slowed down for a certain time [8,9,10].

The widespread and intensive use of polymers in almost all areas of everyday life and industry has significant environmental consequences. The increasing amount of non-biodegradable and toxic plastic waste leads to progressive degradation of the natural environment, and the presence of microplastics in water and food products is becoming a serious threat to both ecosystems and human health. Therefore, the development of a new generation of polymer materials that combine technological functionality with biodegradability and non-toxicity to humans and the environment is becoming increasingly important. The use of such materials, including in membrane technology, can be a key element in strategies to reduce the negative impact of the polymer industry on the environment, while enabling the development of more sustainable solutions in areas such as water treatment, medicine, and the food industry [11,12].

The current production of capillary membranes is growing rapidly, which is a result of their wide and ever-increasing application, especially in drinking water treatment, wastewater treatment, and water recovery technology. Membranes of this type are also finding increasingly wider application in medicine, including in hemodialysis, hemoperfusion, plasma filtration, and artificial organ support systems. High separation efficiency, mechanical properties, and the ability to control porosity make capillary membranes a material of key importance in both the environmental protection sector and modern medicine. The polymers that dominate their production, such as polypropylene (PP) and polyethylene (PE), are characterized by high chemical durability and thus a lack of biodegradability at the end of their life cycle. This results in permanent environmental pollution, microplastic generation, and a growing problem of plastic waste management. In the context of these challenges, it is necessary to develop new biodegradable membrane materials that could reduce the negative impact on the environment while maintaining the required functional properties, especially in medical applications [13,14,15,16].

The first important aim of our research is to find a suitable polymer that will undergo slow degradation. Among several potentially suitable polymers (polylactide [17,18], polycaprolactone (PCL) [19,20], polyglycolide [21], lactide-glycolide-caprolactone copolymer), we chose PCL as a degradable polymer. The advantage of these compounds is that they decompose into simple, non-toxic components without harming the environment. They have been used successfully in medicine for many years, including in surgical sutures, tissue engineering scaffolds, and controlled drug delivery systems. Their ability to decompose in the biological environment into harmless products makes them attractive materials for medical applications, especially temporary implants and devices that come into contact with body fluids [22,23]. Pure PCL is not suitable as the only component of the membrane. It is unknown to what extent and how quickly it will degrade, which will lead to permanent damage to the membrane. Therefore, a second durable and degradation-resistant polymer is necessary to form a durable membrane scaffold. Such a polymer is polyethersulfone (PES), which is chemically resistant and widely used in capillary membranes [24,25,26]. PES has already been successfully used in composite membranes [27,28,29] and even in cellular scaffolds [30]. A necessary condition is good solubility of both polymers in the same solvent and good mutual miscibility.

Biodegradable membranes have already been used in combination with durable polymers as partially degradable composite membranes to reduce fouling. Good results have been obtained for polysulfone(PSf)/lactide-glycolide-caprolactone membranes [31] as well as for PSf/polyurethane (PU) membranes containing, as a degradable polymer, a modified PU containing easily degradable ester bonds [32,33,34].

As part of the work, degradation studies were conducted not only in an alkaline environment (1 molar NaOH solution) but also in simulated body fluid (SBF) with a pH of 7.4, whose ionic composition reflects the conditions prevailing in blood plasma [35]. This allowed us to assess the potential usefulness of these membranes also in biological conditions [36]. The SBF fluid was prepared in the laboratory according to the method described in the literature [37,38].

In an era of growing demand for biocompatible, functional, and environmentally friendly polymer materials for medical applications, the presented research contributes to the development of a new generation of membranes.

## 2. Results and Discussion

### 2.1. Structure of PES/PCL Membranes

**Figure 1:**
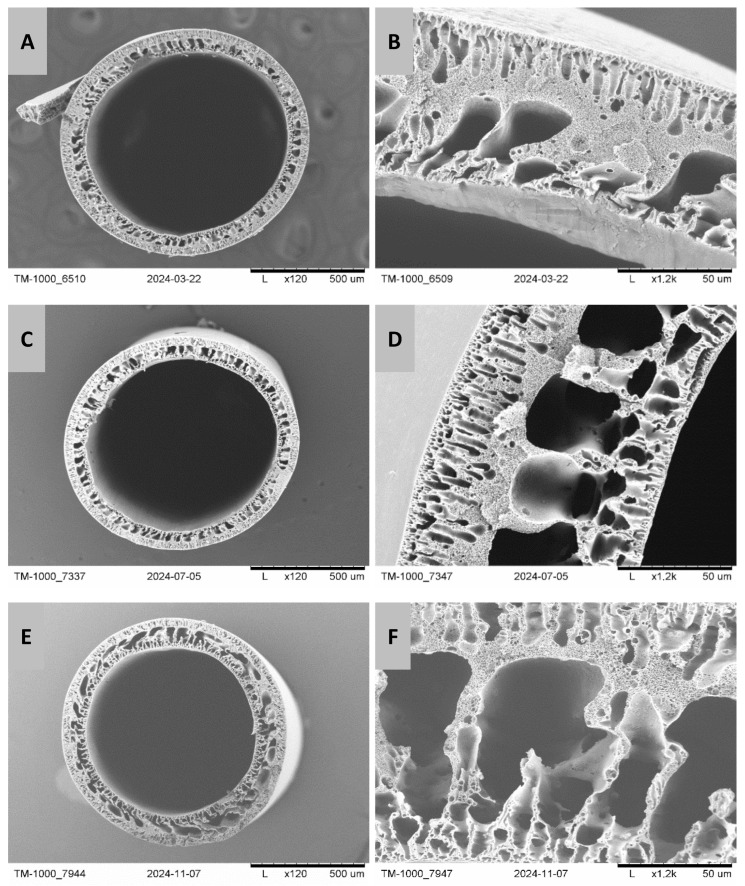
SEM images of PES/PCL 5-1 membranes before and after degradation: (**A**)—membrane structure before degradation, (**B**)—cross-section of the membrane structure before degradation, (**C**)—membrane structure after NaOH degradation, (**D**)—cross-section of the membrane structure after NaOH degradation, (**E**)—membrane structure after SBF degradation, (**F**)—cross-section of the membrane structure after SBF degradation. Scale bars: (**A**,**C**,**E**)—500 µm; (**B**,**D**,**F**)—50 µm.

**Figure 2:**
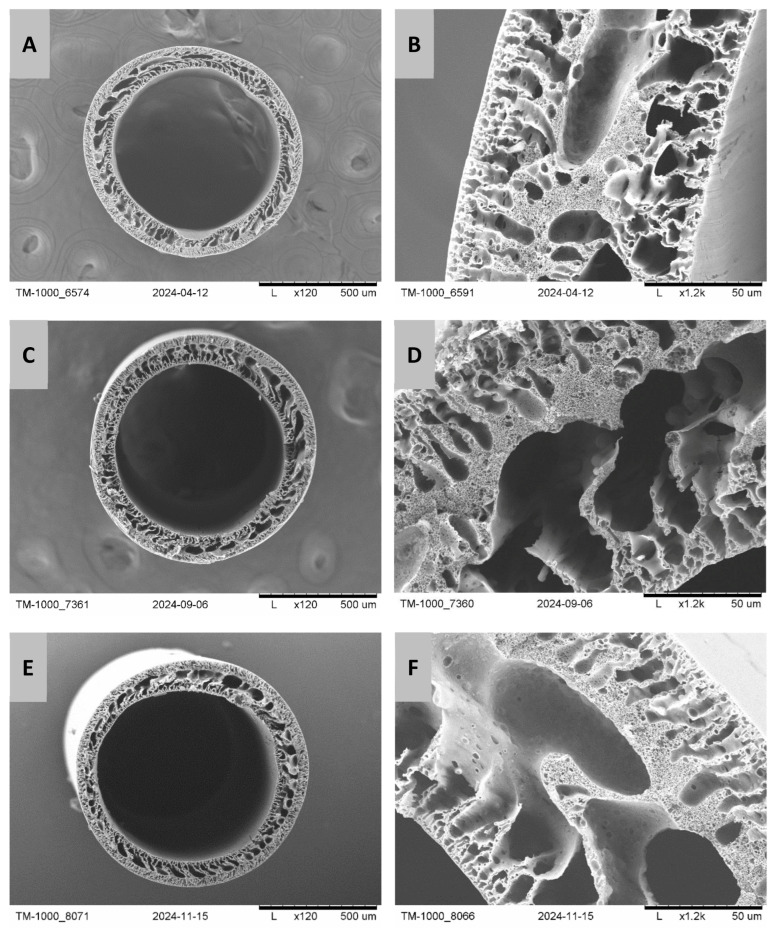
SEM images of PES/PCL 5-3 membranes before and after degradation: (**A**)—membrane structure before degradation, (**B**)—cross-section of the membrane structure before degradation, (**C**)—membrane structure after NaOH degradation, (**D**)—cross-section of the membrane structure after NaOH degradation, (**E**)—membrane structure after SBF degradation, (**F**)—cross-section of the membrane structure after SBF degradation. Scale bars: (**A**,**C**,**E**)—500 µm; (**B**,**F**)—50 µm; (**D**)—100 µm.

In order to illustrate the changes in the structure of PES/PCL membranes before and after degradation with NaOH and SBF solutions, SEM images of the membrane cross-section were taken (Figure 1 and Figure 2). For the PES/PCL 5-1 membrane, the diameter is 815 µm and the wall thickness is 100 µm, while for the PES/PCL 5-3 membrane, the diameter is 720 µm and the wall thickness is 100 µm.

Comparing fragments of membrane wall cross-sections before and after degradation, we can see a difference in membrane structure. Before degradation, the structure of the membranes is denser with a small number of large macropores. However, after degradation, both in NaOH and SBF, the structure becomes looser and more porous. The number of macropores, especially their size, increases. This observation suggests the leaching of part of the membrane material (degradable PCL) from its structure. Large macropores form in place of the removed PCL.

### 2.2. Analysis of Membrane Structure Before and After Degradation Using MeMoExplorer^TM^ Software

**Figure 3:**
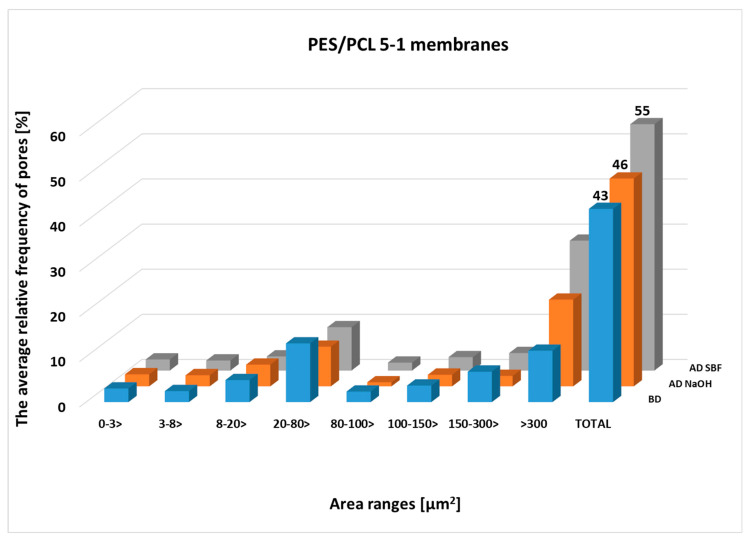
The average relative frequency of pores in eight size classes for the cross-section of the PES/PCL 5-1 membranes and the total area of pores (TOTAL) to the whole SEM image size of the PES/PCL 5-1 membranes before degradation (BD) and after degradation (AD).

**Figure 4:**
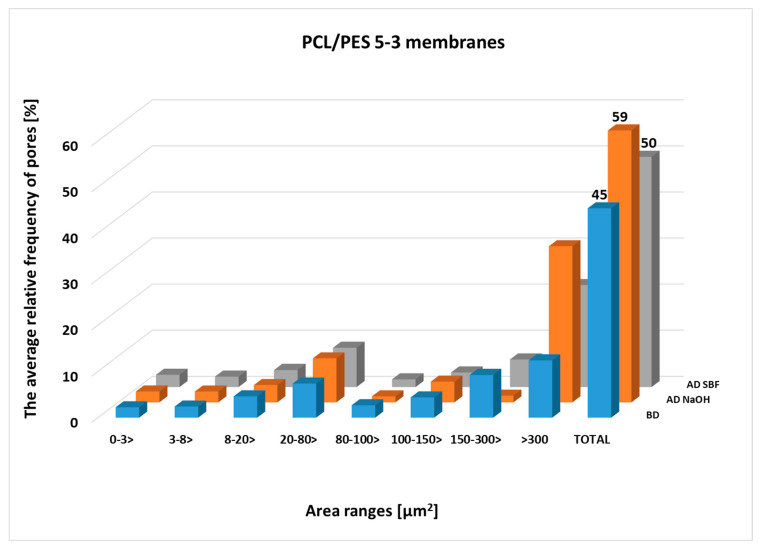
The average relative frequency of pores in eight size classes for the cross-section of the PES/PCL 5-3 membranes and the total area of pores (TOTAL) to the whole SEM image size of the PES/PCL 5-3 membranes before degradation (BD) and after degradation (AD).

Pore size was assessed based on SEM photomicrographs analysis of membranes both before and after degradation in NaOH and SBF. Figure 3 and Figure 4 show the average pore fraction (expressed as a percentage) in eight different size categories for PES/PCL 5-1 and PES/PCL 5-3 membranes, both before and after degradation in NaOH and SBF. The largest pore distribution is in the range above 300 µm^2^. The situation is similar for membranes after degradation, where an increase in pore frequency is noticeable in the range above 300 µm^2^. Also, after degradation in both solutions, there is a noticeable upward trend for pore areas above 300 µm^2^. For the PES/PCL 5-1 membrane, the highest increase (by 10.9%) was after degradation in SBF, while in the NaOH solution, the increase was approximately 1.2%. In the case of the PES/PCL 5-3 membrane, the highest increase in pores was in the range above 300 µm^2^—in NaOH, the increase was 21.5%, while in SBF it was 9.7%. The porosity coefficient (TOTAL) for the membranes before degradation was 42.8% ± 1.99 and 45.4% ±11.06 for PES/PCL 5-1 and PES/PCL 5-3, respectively. After degradation, the highest increase was recorded in SBF to 54.5% ± 9.50 for PES/PCL 5-1. For PES/PCL 5-3, the highest porosity coefficient was recorded after degradation in NaOH solution to 59.0% ± 3.90 (an increase of 13.6%).

The analysis of pore size distribution clearly indicates that degradation in both NaOH and SBF contributes to an increase in the frequency of larger pores (>300 µm^2^). This effect suggests partial leaching of the biodegradable PCL phase from the membrane matrix, which results in structural loosening and higher overall porosity. The differences observed between PES/PCL 5-1 and PES/PCL 5-3 compositions confirm that the PCL content strongly influences the extent of structural changes. In particular, the more pronounced increase in pore fraction in NaOH for PES/PCL 5-3 (21.5%) reflects the higher susceptibility of membranes with greater PCL content to hydrolytic degradation. On the other hand, the relatively higher porosity increase in PES/PCL 5-1 in SBF indicates that even at lower PCL content ionic components of simulated body fluid can accelerate the degradation process.

The increase in the porosity coefficient after degradation (up to 59.0% for PES/PCL 5-3) may have practical implications, as it can improve membrane permeability and mitigate pore blockage by reducing fouling. At the same time, the stable PES component ensures mechanical integrity and prevents excessive disintegration of the structure. This complementary effect highlights the potential utility of PES/PCL membranes in both environmental and biomedical applications.

### 2.3. Instability Coefficient of Membranes Before and After Degradation

**Figure 5:**
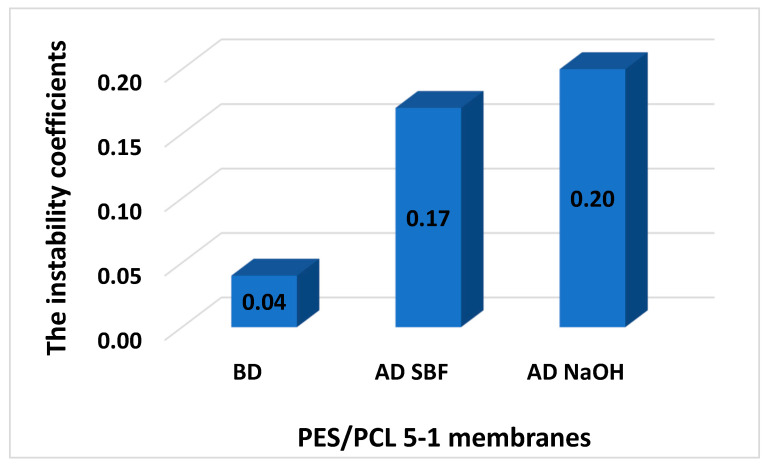
The instability coefficients of membranes, before degradation (BD) and after degradation (AD) of PES/PCL 5-1 membranes.

**Figure 6:**
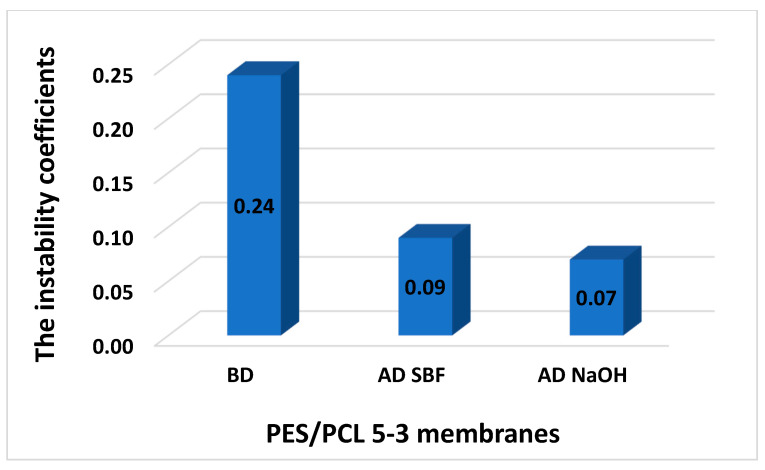
The instability coefficients of membranes, before degradation (BD) and after degradation (AD) of PES/PCL 5-3 membranes.

The results of the instability coefficient are presented in Figure 5 and Figure 6. The diagram shows the instability coefficients of the membranes before and after degradation in NaOH solutions and SBF.

The PES/PLC 5-1 membrane achieved better results before degradation compared to the PES/PCL 5-3 membrane. In each case, a good stabilizing effect was observed for both membranes before and after degradation. It was generally low and did not exceed 0.25, which indicates the repeatability of the membranes.

A low instability coefficient indicates high repeatability of the membrane production process, which means that the membrane parameters are stable and homogeneous.

Membranes with an instability coefficient below 0.1 are characterized by high stability and repeatability. Values in the range of 0.25–0.30 can be considered (based on our experience to date) as the limit of acceptable repeatability, as the differences in porosity are not significant. An instability coefficient above 0.3 may suggest excessive deviations and a lack of repeatability of the membrane parameters.

The obtained instability coefficients confirm that both PES/PCL 5-1 and PES/PCL 5-3 membranes were produced with high reproducibility, as all values remained well below the threshold of 0.25. This demonstrates that the introduction of the biodegradable PCL phase did not compromise the homogeneity of the membrane structure, even after exposure to degradation in NaOH or SBF. The slightly better stability observed for PES/PCL 5-1 suggests that a lower PCL content may favor more uniform membrane formation, while higher PCL loading (PES/PCL 5-3) still remained within the acceptable range of repeatability. Overall, the low instability coefficient values confirm the reliability of the fabrication process and support the potential for scaling these membranes to practical environmental and biomedical applications.

### 2.4. Transport and Separation Parameters of Membranes Before and After Degradation

#### 2.4.1. Hydraulic Permeability of Membranes (UFC)

**Figure 7:**
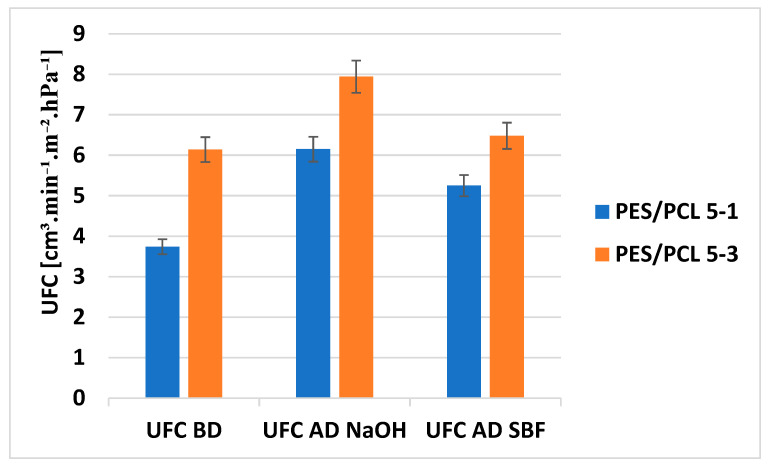
UFC [cm^3^∙min^−1^∙m^−2^∙hPa^−1^] membranes before degradation (BD) and after degradation (AD).

The UFC of the membranes was measured before and after degradation with NaOH and SBF solutions. UFC values or the PES/PCL 5-1 membrane are marked in blue, and for the PES/PCL 5-3 membrane in red. The measurement results are presented in Figure 7.

For the PES/PCL 5-1 membrane. UFC increases from 4.63 to 6.15 after degradation with NaOH solution and to 5.25 after SBF solution. Similarly, for the PES/PCL 5-3 membrane, UFC increases from 6.14 to 7.94 after degradation with NaOH solution and to 6.48 after SBF solution. The UFC for the PES/PCL 5-1 membrane increases by 33% after NaOH degradation and by 13% after SBF. Similarly, UFC for PES/PCL 5-3 membrane increases by 29% after NaOH degradation and by 6% after SBF. Thus, NaOH digestion causes a greater increase in UFC than SBF digestion. Both after the digestion of NaOH and SBF, membranes with greater porosity than the original membranes were obtained. The looser structure and greater porosity lead to higher UFC of the membranes, resulting in increased flow efficiency of the solutions filtered through the membrane.

#### 2.4.2. Separation Properties of Membranes

Retention coefficients were determined for the PES/PCL membrane before and after degradation with NaOH and SBF solutions on selected markers. The following compounds were used as markers:

- PEGs with an average molecular weight of 4000 g mol^−1^ [4 kD], 15,000 g mol^−1^ [15 kD], 35,000 g mol^−1^ [35 kD].
- Chicken egg albumin (CEA) with an average molecular weight of 45,000 g mol^−1^ [45 kD].
- Bovine albumin (BSA) with an average molecular weight of 67,000 g mol^−1^ [67 kD].

**Figure 8:**
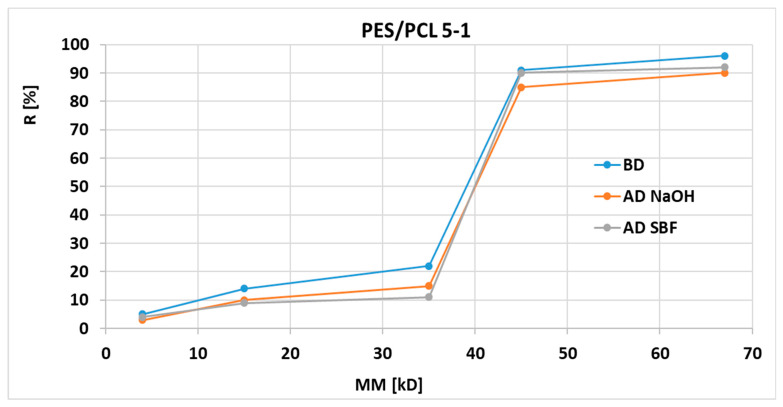
Retention coefficient values for different markers obtained for PES/PCL 5-1 membranes before degradation (BD) and after degradation (AD).

**Figure 9:**
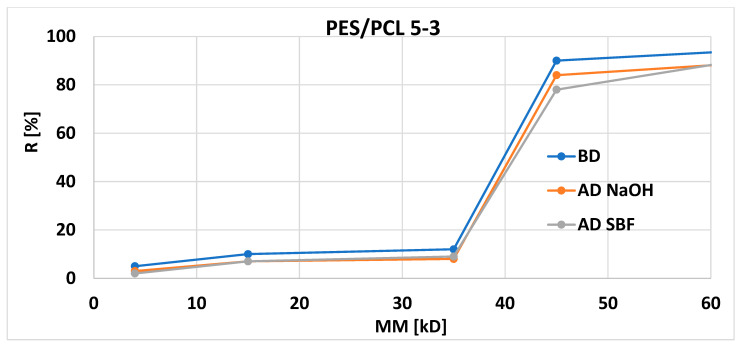
Retention coefficient values for different markers obtained for PES/PCL 5-3 membranes before degradation (BD) and after degradation (AD).

Graphs of the dependence of the PES/PCL membranes retention [R] on the molecular weights [MW] of the markers used before and after degradation are shown in Figure 8 and Figure 9 (blue before degradation, red after NaOH degradation, gray after SBF degradation).

The retention coefficients of PES/PCL 5-1 and PES/PCL 5-3 membranes after degradation of the membranes with NaOH and SBF solutions do not show significant differences compared to the base membrane. We observe a small but noticeable decrease in retention for membranes after degradation (in NaOH and SBF) for all markers used, which is consistent with the expected results.

### 2.5. Changes in PCL Content in Membranes After Degradation

In order to determine the extent to which PCL is eliminated from the PES/PCL membrane structure. The mass of PCL in the membranes before and after degradation was measured. By measuring the mass of membranes before and after degradation and knowing the percentage of PCL in each membrane. One can easily determine the degree of PCL removal from the membrane after degradation. The measurement results are presented in Table 1. It was assumed that the PES content in the membranes remains unchanged.

The degree of PCL removal from the PES/PCL 5-1 membrane after NaOH degradation is from 37 to 40%, and for the PES/PCL 5-3 membrane, it is from 66 to 71%. In the case of PES/PCL 5-1 membranes after SBF degradation, the PCL removal rate ranges from 47 to 52%, and for PES/PCL 5-3 membranes it ranges from 48 to 52%. The mass loss of PCL from membranes after degradation is significant and amounts to approximately 50% on average. The largest mass loss of PCL is observed for the PES/PCL 5-3 membrane, which is expected because this membrane contains more PCL in its structure. Both the NaOH solution and SBF have proven to be effective etching agents, allowing the production of membranes with high porosity.

## 3. Materials and Methods

### 3.1. Materials

PES (Ultrason E2020P. BASF. Mw = 42,000 g mol^−1^); PCL (Sigma Aldrich, St. Louis, MO, USA. Mw = 45,000 g mol^−1^); N-methyl-2-pyrrolidone (NMP) (Sigma Aldrich, St. Louis, MO, USA); Polyethylene glycols (PEG) (Fluka. Neu-Ulm, Germany. Mw = 4000; 15,000; and 35,000 g mol^−1^); Chicken egg albumin (CEA) (Fluka. Germany. Mw = 45,000 g mol^−1^); Bovine serum albumin (BSA) (Fluka. Germany. Mw = 67,000 g mol^−1^); Sodium hydroxide (NaOH) (POCH, Gliwice, Poland); Simulated body fluid (SBF); Glycerin (POCH, Gliwice, Poland) was dried overnight under vacuum at 100 °C before usage; NMP was used as the solvent for PES and PCL; NMP was dried with a molecular sieves 4A (POCH) before usage.

### 3.2. Preparation of Capillary Membranes

PES was selected as the base polymer, and PCL as the biodegradable polymer for the fabrication of capillary membranes. Both compounds were first dissolved separately in NMP at 25 °C for 24 h, each in a separate vessel, until complete dissolution. Subsequently, the two polymer solutions were combined and mixed for an additional 24 h using a magnetic stirrer to ensure homogeneity The concentration of the polymer mixture (PES and PCL together) in the solvent was constant each time and amounted to 20% by weight.

### 3.3. Capillary Membrane Fabrication

Capillary membranes were fabricated using a dedicated production facility. The membranes were produced via a dry/wet spinning phase-inversion method, involving extrusion of a polymer solution. Their formation process was monitored and controlled using specialized software, which allowed for rapid adjustment of operational parameters. Spinning was carried out at ambient temperature. Subsequently, the membranes were cut into small sections and immersed in a clean water bath for four days to remove residual solvents. Following the washing step, the membranes were stabilized in a 10% aqueous glycerine solution for one day. The residual glycerine retained within the membrane pores enhanced their hydrophilicity. Subsequently, the capillaries were air-dried at ambient temperature. Capillary membrane modules were assembled by placing twenty capillary segments, each 6 cm in length, into a polypropylene housing with an approximate surface area of 25 cm^2^. Epoxy resin was employed to hermetically seal both ends of the module. Two types of membranes were obtained with the PES/PCL weight ratio of 5-1 and 5-3, respectively. They are marked PES/PCL 5-1 and PES/PCL 5-3.

### 3.4. SEM Analysis

The morphology of the fabricated membranes was examined using scanning electron microscopy (SEM) with a Hitachi TM-1000 instrument (Chiyoda-ku, Japan). SEM samples were prepared by fracturing the membranes in liquid nitrogen to prevent structural deformation during breakage. Subsequently, the samples were coated with a 10 nm gold layer using a sputter coater (EMITECH K 550X, Quorum Technologies, East Sussex, UK).

### 3.5. Permeability Measurements

Hydraulic permeability was assessed by measuring the volume of solution permeating through the membrane walls over a 10 min period under a transmembrane pressure of 200 hPa. The UFC was calculated in accordance with Equation (1).

*UFC = V/(T · P · p)* (1)

where v—volume of the solution (cm^3^), t—time of measure (min.), a—nominal capillary area in module (m^2^), and p—transmembrane pressure (hPa).

### 3.6. Retention Measurements

The retention coefficient describes the separation performance of the membrane. Membrane retention, R (%), is defined according to Equation (2).

*R* = {1 − (*C_P_/C_n_*)} 100% (2)

where R—retention coefficient, C_P_—concentration of marker in permeate (g dm^−3^), and C_R_—concentration of marker in retentate (g dm^−3^).

PEG with molecular weights of 4, 15, and 35 kDa, chicken egg albumin (CEA), and bovine serum albumin (BSA) were successively used to evaluate the retention of each capillary module. Marker concentrations were determined using a UV spectrophotometer (Hitachi U-3010) at a wavelength of 190 nm for PEG and 280 nm for CEA and BSA.

### 3.7. Membrane Degradation

An aqueous solution of 1 molar NaOH in the amount of 1 dm^3^ for each module was passed through modules with PES/PCL 5-1 and PES/PCL 5-3 membranes. 6 modules were made for each membrane. The etching solution passed through the membrane walls from the inside to the outside of the membrane. The digestion process was carried out in a closed circuit for 7 days. Analogous degradation was performed using an SBF solution. In this case. The digestion process lasted 21 days for each module.

### 3.8. Evaluation of SEM Photomicrographs

Membrane morphology was analyzed with the aid of MeMoExplorer^TM^ software. The study analyzed at least 5 SEM photomicrographs of membrane cross-sections (PES/PCL 5-1 and PES/PCL 5-3) both before and after degradation in SBF and NaOH. SEM images were taken at 1200× magnification. MeMoExplorer^TM^ software was then used for analysis, which included determining pore contours and calculating their surface area. It is worth noting that this program can classify pores into 8 different size classes, as shown in Table 2. In addition, it allows the calculation of the porosity coefficient (Total). The porosity coefficient (Total) is the percentage of all pores of the entire size of the SEM image.

### 3.9. Assessment of the Membrane Instability Coefficient Before and After Degradation

The MeMoExplorer^TM^ software allows data to be prepared for further statistical analysis, which can be performed using any external tools (e.g., Statistica, Origin, or Excel). In the conducted research, Excel was used to determine the average values of porosity coefficients (Ave) and standard deviations of porosity coefficients (DS). Based on the data obtained, the instability coefficient of the membranes was determined. The instability coefficient is a measure of the repeatability of membrane parameters obtained in the production process. It is calculated as the ratio of the standard deviation (SD) of the porosity coefficient to its average value (Ave), according to Formula (3).

*Instability coefficient = SD/Ave* (3)

where

- SD—Standard deviation of the porosity coefficient.
- Ave—Average of the porosity coefficient.

### 3.10. Statistical Analysis

All the quantitative data were obtained from at least five samples for analysis. Results were expressed as the average ± standard deviation (Ave ± SD).

## 4. Summary and Conclusions

The subject of this work was to obtain semipermeable two-component blend capillary membranes PES/PCL, where one component, PCL, would gradually undergo partial degradation/hydrolysis during membrane operation. Two types of membranes were made, PES/PCL 5-1 and PES/PCL 5-3, differing in the content of PCL in the membrane. The membranes were subjected to degradation by 1 molar solutions of NaOH and SBF through them.

The percentage loss of PCL after hydrolysis was determined, which on average amounted to about 50% of the PCL mass loss. The best degradation result was achieved for the PES/PCL 5-3 membrane etched with NaOH solution, which amounted to about 70%. In all cases, after partial degradation of PCL, the UFC of the membranes increased. After NaOH etching, the UFC increase was greater than after SBF etching. The retention coefficient values and the limiting molar masses of the obtained membranes were determined. For both types of membranes, after NaOH and SBF etching, slightly lower retentions were obtained for all the markers used.

Based on SEM images, the structures of membranes before and after degradation were determined and compared. The structure of membranes at the surface is compact, with a spongy structure, and contains finger-like macropores. While inside the capillary walls, the structure is looser and has cavernous macropores. The structure of membranes after NaOH and SBF hydrolysis is looser, with enlarged macropores. This was also confirmed using the analysis using the MeMoExplorer^TM^ software. Moreover, the work showed a low coefficient of membrane instability before and after degradation, which indicates that a repeatable membrane structure was obtained.

PES/PCL membranes containing degradable PCL can be useful in long-term separation processes. Hydrophobic PES membranes are susceptible to fouling, which leads to a decrease in the efficiency of membrane processes. To reduce this unfavorable phenomenon, a hydrophilic PCL polymer (containing degradable ester bonds) is added to the membrane-forming solution, which will undergo partial degradation under the influence of slow hydrolysis during membrane operation. Thanks to this, the porosity and hydraulic permeability of the membranes will increase, without causing a decrease in their separation efficiency despite the occurring fouling. Increasing the porosity of the membranes is an effective way to reduce the fouling phenomenon and extend their operating time.

## Figures and Tables

**Table 1:** PCL mass removed from PES/PCL membranes after degradation in NaOH and SBF.

Module	Mass of Module with Membranes Before Degradation [g]	Mass of Membranes in the Module Before Degradation [g]	PCL Mass in MEMBRANES Before Degradation [g]	Mass of Module with Membranes After DEGRADATION [g]	Mass of PCL Removed After Degradation [g]	PCL Mass Removed After Degradation [%]
PES/PCL 5-1				NaOH	NaOH	NaOH
1	3.7963	0.0925	0.0154	3.7901	0.0062	40
2	3.8061	0.0942	0.0157	3.8003	0.0058	37
3	3.7493	0.0959	0.0160	3.7432	0.0061	38
				SBF	SBF	SBF
1	3.7544	0.0994	0.0166	3.7458	0.0086	52
2	3.7019	0.0977	0.0163	3.6942	0.0077	47
3	3.6807	0.0994	0.0166	3.6726	0.0081	49
PES/PCL 5-3				NaOH	NaOH	NaOH
1	3.6758	0.0939	0.0352	3.6519	0.0239	68
2	3.8175	0.0922	0.0346	3.7929	0.0246	71
3	3.7123	0.0939	0.0352	3.6891	0.0232	66
				SBF	SBF	SBF
1	3.7525	0.0906	0.0340	3.7362	0.0163	48
2	3.4628	0.0988	0.0371	3.4442	0.0186	50
3	3.6710	0.0939	0.0352	3.6527	0.0183	52

**Table 2:** Pore size classes in the MeMoExplorer^TM^ program.

Pore Size Class	1	2	3	4	5	6	7	8	9
Size [µm^2^]	0–3	3–8	8–20	20–80	80–100	100–150	150–300	>300	Total

## Data Availability

No new data were created or analyzed in this study. Data sharing is not applicable to this article.
